# Challenging the ADHD consensus

**DOI:** 10.3402/qhw.v11.31124

**Published:** 2016-04-05

**Authors:** Soly Erlandsson, Elisabeth Punzi

**Affiliations:** Department of Social and Behavioral Studies, University West, Trollhättan, Sweden; Department of Psychology, Gothenburg University, Gothenburg, Sweden

Psychiatric diagnoses are based on a classification system, which not only builds on biomedical facts but which is also influenced by a wide array of political, economic, and professional interests (see, e.g., Frances & Widiger, 2012; Leo & Lacasse, 2015). In the case of Attention-Deficit Hyperactivity Disorder (ADHD), the vast majority of resources financially and professionally support the biomedical model for diagnosing children and adults with ADHD-like behavior. It is also easier for researchers to receive financial support for studies on ADHD if they engage in the neurobiological field (Goldfried, 2015), which is conducive for the pharmacological industry to develop new medical compounds for the treatment of ADHD. In today's complex and multicultural society, however, we believe it is not enough to embrace one model—the biomedical—to understand aberrant human behaviors.

Criteria for an ADHD diagnosis as well as names of pharmaceuticals to remedy the disorder are readily available on the Internet (Pedersen, 2015; Vrecko, 2015). However, researchers as well as clinicians have raised concern that stimulant prescription to children is on the increase although long-term risks and benefits are unknown at the present time (see, e.g., LeFever Watson, Arcona, Antonuccio, & Healy, 2014). At the same time, vulnerable young people might look for solutions to their hardships on chat rooms full of naïve ideas about the advantages of being diagnosed with ADHD. Acknowledging those risks it is our duty, as researchers and clinicians, to also reflect on the ways in which social dilemmas and an insecure life situation caused, for instance, by the loss of a close family member, parents’ divorce or economic hardship, might influence the child's well-being and behavior. But not only reflect—we need to take those aggravating circumstances into consideration when trying to comprehend and care for a child who suffers. *Children may behave hyperactively as a response to basic emotional needs not being filled or as a reaction to overstimulation, and their aberrant behavior should thus be seen as a form of communication and not as a mere symptom of a biomedical disease*. By choosing one single biomedical code, the “true” story will never be heard.

Diagnoses such as depression and substance use disorders are increasingly classified as neurological disorders or conditions, implying that there is a known neurobiological dysfunction (Leo & Lacasse, 2008; Vrecko, 2010). Even though researchers from various disciplines have shown that it is inadequate to view ADHD as a neurobiological disorder, surprisingly little criticism has been directed toward the biomedical explanation in clinical practice or in the media. In popular media, for example, so-called neuropsychiatric diagnoses have been presented as severe threats to public health (Börjesson, 1999). The hegemonic status of the current medical discourse on ADHD reflects some kind of social consensus. In line with this hegemony, even teachers are encouraged to “discover” children who might suffer from ADHD. Human suffering, however, tends to be complex, and a purely neurobiological discourse focused on diagnostic criteria downgrades the importance of contextual factors such as socioeconomic impact and exposure to mistreatment. Thereby, the complex needs and interests of the individuals concerned are not taken into consideration. Instead, according to Laclau and Mouffe (1985), peoples’ interests and needs are masked in a discourse where social consensus is prevalent.

So, we need to ask ourselves: Can we, by interrogation and observation, approach the masked needs and interests of children that are now diagnosed with ADHD? It might well be the case that the parent of “the problem child” is the one who foremost needs help and support. Francoise Dolto, the French child psychiatrist and psychoanalyst (1908–1988) once said that the parent who is deeply bothered by his/her child's behavior is the one who needs treatment. Today, shifting the focus from the child to the parents is, however, almost perceived as a threat not only to the parents but—ironically—also to the experts on ADHD. It is not the parents’ fault that their child is acting divergently. Such behavior problems in the child can, however, be linked to an unbalanced situation in the family and to the family history. Instead of examining the family dynamics and masked dysfunctions in parents, it is of course less complicated to put the blame on the child. The tendency to diagnose human suffering as a biomedical disorder might also lead to the marginalization of certain groups of people. Frances and Widiger (2012) argue that “the greater the number of health clinicians, the greater the number of life conditions that work their way into becoming disorders” (p. 111). The window to “normality” might reach a point where it becomes hard for anyone to squeeze in.

It is remarkable that researchers and practitioners from various professions so easily seem to accept the biomedical model of ADHD and perceive pharmacological solutions as appropriate. When complicated human conditions are presented as defined categories, and when questionnaires and diagnostic criteria are perceived as appropriate responses to human suffering, it is necessary to reflect on alternative models and interventions. Qualitative studies have the capacity to acknowledge complexities and paradoxes as well as contextual factors, and thereby challenge hegemonic systems of classification. Qualitative studies may also provide insight into the complex processes and experiences that underlie aberrant behaviors. We therefore look forward to alternative perspectives and critical investigations of the current hegemonic view on children who are perceived as restless, inattentive, and/or impulsive. You are welcome to submit your work to *International Journal of Qualitative Studies on Health and Well-being*.

*Soly Erlandsson*, Professor Department of Social and Behavioral Studies University West Trollhättan, Sweden Email: soly.erlandsson@hv.se *Elisabeth Punzi*, PhD Department of Psychology Gothenburg University Gothenburg, Sweden
